# Comprehensive Evaluation of Rice Qualities under Different Nitrogen Levels in South China

**DOI:** 10.3390/foods12040697

**Published:** 2023-02-06

**Authors:** Chao Ding, Congshan Xu, Bo Lu, Xuhui Zhu, Xikun Luo, Bin He, Cambula Elidio, Zhenghui Liu, Yanfeng Ding, Jie Yang, Ganghua Li

**Affiliations:** 1 Institute of Food Crops, Jiangsu Academy of Agricultural Sciences, Nanjing 210014, China; 2 Jiangsu Collaborative Innovation Center for Modern Crop Production, National Engineering and Technology Center for Information Agricultrue, Key Laboratory of Crop Physiology and Ecology in Southern China, Nanjing Agricultural University, Nanjing 210095, China

**Keywords:** indica rice, japonica rice, nitrogen, comprehensive quality, principal component analysis

## Abstract

There is a need to comprehensively evaluate the rice quality of different rice varieties under different nitrogen treatments. Therefore, in this study, we used twenty-one hybrid indica rice varieties and twenty-three inbred japonica rice varieties with three nitrogen fertilizer levels to investigate differences in rice qualities. As compared with hybrid indica rice, inbred japonica rice had lower coefficient of variation values for grain shape, mild rice percentage, and head rice percentage, but relatively higher coefficient of variation values for chalkiness traits, appearance, and taste value of cooked rice. A principal component analysis and membership function method were used to comprehensively evaluate the qualities of rice. The overall eating quality value by sensory evaluation and head rice percentage explained 61.3% and 67.9% of the variations in comprehensive quality of hybrid indica rice and inbred japonica rice across different nitrogen levels, respectively. We also found that rice comprehensive quality was better under low nitrogen levels for hybrid indica rice, while for inbred japonica rice, properly increasing nitrogen application could improve the comprehensive quality.

## 1. Introduction

China is one of the largest rice production and consumption countries in the world. According to the National Statistical Yearbook of 2021 [1], in 2021, the rice planting area in China was about 29.9 million, which ranked China second in the world, with a total output of about 213 million tons, which ranked China first in the world. The average yield per ha has been reported to be 7.1 tons, which is 1.71 times that of the world level. Although China’s rice output has been stable at more than 200 million tons for 11 consecutive years, the demand for high-quality rice is relatively high, and the import volume remains to be 2–5 million tons year after year. This highlights the current structural contradiction between production and demand of rice in China. In order to solve the problem of food and clothing, for a long period of time, the goal of rice breeding and cultivation in China has mainly focused on yield increase, which has led to low-quality rice. In recent years, Chinese scientists have shifted the goal of rice breeding to equal emphasis on yield and quality. 

Rice produced in the north, especially in Northeast China, has the characteristics of high quality and fragrant taste, due to the fertile soil, good water quality, and large temperature difference between day and night [2,3]. In South China, hybrid indica rice has high yield but low eating quality, and the appearance and taste quality of japonica rice are relatively poor because of the high temperature and humidity environment [4]. In the past 20 years, driven by innovative technologies in rice breeding scientific research, the process of high-quality rice breeding in South China has been significantly accelerated [5,6]. A number of high-quality rice varieties with good taste have emerged. Representative high-quality indica rice varieties are Yixiangyou 2115, Chuanyou 6203, Mei Xiangzhan, and Fengyou Xiangzhan; representative high-quality japonica rice varieties are Nanjing 46, Nanjing 9108, Ningjing 8, and Ningxiangjing 9.

Rice quality mainly includes milling quality, appearance quality, and eating quality. The milling and appearance quality of rice can be quantitatively evaluated according to the standard of high-quality rice (GB/T17891-2017) [7]. The evaluation of eating quality is the most difficult, and there are many methods for evaluating the taste quality of rice. The first method is sensory evaluation which involves directly scoring, according to the corresponding standards, the appearance, smell, taste, texture, retrogradation, and overall eating quality of rice [8]. Although sensory evaluation is time-consuming and laborious, this method can accurately evaluate the eating quality of varieties and can directly reflect consumers’ taste preferences; therefore, it has been widely studied and used. The second method is the instrument method; the principle of a taste analyzer used at present is mostly based on the reflection of near-infrared and visible light and the determination of the physical and chemical properties of rice, combined with a mathematical model that is established using sensory scores with the help of computer software to predict the taste of rice [9]. The advantages of the instrument method are the small sample size, and the simple/fast operation; the disadvantages of the instrument method are that the taste meter is not applicable to different regions and indica rice varieties, and the taste meter cannot evaluate the smell. The third method is the physical and chemical index method; most studies have shown that rice with good taste has lower amylose content, lower protein content, higher fat content, lower gelatinization temperature, and longer gel consistency [10,11,12]. However, with more in-depth research, scholars have found that it was difficult to accurately reflect the eating quality of rice by only using the above physical and chemical properties [13,14]. There have also been studies that have combined the eigenvalues of RVA spectra of rice with the texture properties of rice to evaluate the eating quality of rice. Chen et al. [15] found that protein and protein plus amylose explained 38.6% and 62.1%, respectively, of the variation in taste value of indica rice in Southwest China; the RVA value explained 60.5% of the variation. 

However, the taste of rice in a country or region cannot be covered and explained by rice classification standards and physical and chemical indexes. There are many and complex types of rice varieties grown in China, and significant differences in eating habits have been observed between the south and the north, and between urban and rural areas. Since requirements for eating quality are diverse and complex, it is difficult to form a unified standard. Therefore, evaluating rice taste in China should be based on sensory evaluation, supplemented by physical and chemical index detection.

In order to improve the breeding efficiency of high-quality varieties, there is an urgent need for a method to comprehensively evaluate rice quality. Zhu et al. [16] comprehensively considered eating quality, milling quality, and appearance quality, and established a comprehensive index model of indica rice quality in Hubei and Jiangxi by using principal component analysis. Jiang et al. [17] comprehensively evaluated the rice quality of 14 newly bred rice varieties in Hunan Province, from 2005 to 2006. On the basis of principal component analysis, 18 rice varieties bred in Heilongjiang and Japan were comprehensively appraised by membership function method [18]. The middle and lower reaches of the Yangtze River are suitable for planting both indica and japonica rice, but there is still a lack of comprehensive evaluation of rice quality in this area.

In addition, nitrogen fertilizer is one of the most important factors that affects the quality of rice. Most previous studies have reported that nitrogen fertilizer improved the milling quality and appearance of rice, and reduced the eating quality of rice [19,20]. However, current research studies have mostly focused on the effect of nitrogen fertilizer on individual quality, and few varieties have been selected; therefore, the research results may not have been representative; in addition, the research results have differed because of differences in varieties and environments [21,22], and the lack of comprehensive evaluations on the effects of nitrogen management on rice qualities.

The average nitrogen input for the high-yield area of the middle and lower reaches of the Yangtze River has been reported to be 300 kg ha^−1^. Therefore, in this study, for the field experiment, forty-four rice varieties that are widely grown in South China were subjected to three nitrogen fertilizer levels (0 kg ha^−1^, 150 kg ha^−1^, and 300 kg ha^−1^). The objectives of this study were to explore the quality differences among varieties and the effect of nitrogen fertilizer on the comprehensive quality of different varieties, and therefore, to provide good suggestions for the breeding of high-quality rice and corresponding cultivation techniques.

## 2. Materials and Methods

### 2.1. Experimental Cultivars and Site

Twenty-one hybrid indica rice varieties and twenty-three inbred japonica rice varieties, bred in South China, were selected as experimental materials in 2018. The characteristics of the selected varieties are listed in Table S1. This study was conducted at Danyang County (32°00′ N, 119°32′ E, 7 m a.s.l.), Jiangsu province, China, which is a typical rice-wheat rotation region that is suitable for planting both indica rice and japonica rice. The soil in the upper 20 cm at the experimental site had the following properties: pH 6.7, 21.09 g kg^−1^ organic matter, 85.40 mg kg^−1^ alkali-hydrolysable N, 13.33 mg kg^−1^ available P, and 119.41 mg kg^−1^ available K. 

### 2.2. Experimental Design

The experiment was arranged in a split-plot design with N treatments as the main plots and varieties as the subplots. The three N treatments were as follows: 0 kg ha^−1^ (N0), 150 kg ha^−1^ (N150), and 300 kg ha^−1^ (N300). N fertilizer was applied at the basal, tillering, and panicle initiation stages at a ratio of 4:3:3. Phosphorus (90 kg ha^−1^ as single superphosphate) was applied 1 day before transplanting and potassium (120 kg ha^−1^ as KCl) was applied in two splits: 50% applied 1 day before transplanting and 50% at the panicle initiation stage. The experiment was replicated three times, in 45 m^2^ subplots. Pregerminated seeds were sown in a seedbed at 50 g plot^−1^ and 100 g plot^−1^ for hybrid indica rice and inbred japonica rice, respectively. Seedlings were machine transplanted at 15 days old to field plots, with 17 × 30 cm hill spacings for hybrid indica rice and 12 × 30 cm hill spacings for inbred japonica rice. The transplantation date was June 16. Crop management followed standard cultural practices. Insects were intensively controlled with chemicals to avoid biomass and yield losses.

### 2.3. Evaluation of Appearance Quality and Milling Quality

Grains harvested from each plot were dried until the moisture of the grain was 14%, and then stored at 4 °C for three months. The rice milling and appearance quality analysis was performed according to the GB/T 17891-2017 [7]. The brown rice percentage, milled rice percentage, and head rice percentage were expressed as percentages of the total grain weights. Milled rice grain appearance, including grain length, width, chalkiness rate, chalkiness size, and chalkiness degree were evaluated using a rice appearance quality analyzer (SC-E, Hangzhou Wanshen Test Technology Corporation, Hangzhou, China). 

### 2.4. Sensory Evaluation of Eating Quality of Rice

A sensory panel of 15 trained panelists, who were experts engaged in rice variety selection, production, marketing, and taste hobby, were selected for the evaluation of sensory properties. The number of samples of rice for each test was 10 including the standard. Hybrid indica rice was treated with Liangyoupeijiu under N0 level as standard, and japonica rice was treated with Wuyujing 3 under N0 level as standard. Ten rice cookers were used, with the same model type (MB-FZ40Q, Midea Group, Nanjing, China). Portions (500 g) of milled rice were washed three times. After washing, 1.4 times of water was added for hybrid indica rice and 1.2 times of water was added for inbred japonica rice. The rice samples were soaked for 30 min, and after soaking, the rice samples were cooked automatically using the “perfect cooking program” of the cooker, and held for 10 min after cooking. 

The textural properties of the rice samples, which included appearance, smell, taste, texture, retrogradation, and overall eating quality, were evaluated by the panelists. Each index of rice sample was compared with the control, and the scores were: quite poor, poor, slightly worse, the same as the standard, slightly better, better, and best, which were scored as −3, −2, −1, 0, 1, 2, and 3, respectively. The score of the standard was 70; the taste value of an evaluated rice sample = 70 + average value of evaluated rice sample × 10. The appearance was judged according to the whiteness, luster, structure of rice grains (integrity, extensibility, swelling and deformation, broken grains), degree of embryo retention, miscellaneous color, etc. The taste of the rice was assessed according to the stickiness, hardness, and elasticity of teeth when chewing rice. The texture was evaluated according to whether there was a clear smell and sweetness when chewing rice, and a smooth feeling in the throat when swallowing. The retrogradation was the degree to which the rice became “hard” after it has been kept at room temperature for a certain period of time. Overall eating quality was judged according to a comparison between the comprehensive taste of the evaluated samples and the standard. Three replicates per sample were evaluated for one panelist [23].

### 2.5. Statistical Analyses

The tables were processed using Microsoft Excel 2016 (Microsoft, Redmond, WA, USA). The SPSS 18.0 software (SPSS, Inc., Chicago, IL, USA) and the Origin 2021 software (OriginLab, Northampton, MA, USA) were used for the data processing, two-way analysis of variance (ANOVA), Tukey’s HSD test, mapping, cluster analysis, and principal component analysis (PCA). All significant differences were conducted at *p* < 0.05. 

A PCA can convert multiple indicators into multiple comprehensive indicators to determine their relative importance, and to ensure that the amount of information after dimensionality reduction is maintained at a sufficiently high level. The KMO test was performed during the principal component analysis [24]. The KMO test was mainly used for the data of principal component extraction. Generally speaking, the KMO test coefficient values were distributed between 0 and 1. If the coefficient value was greater than 0.5, the sample was considered to meet the requirements of reasonable data structure. The dimension of 15 quality indexes was reduced by principal component analysis, and then the comprehensive evaluation D value was calculated according to the membership function method and calculated formulas were as follow [24]:

$$U\left(X_{j}\right)=\left(X_{j}-X_{\min}\right)/\left(X_{\max}-X_{\min}\right)$$

where X_j_ is the jth comprehensive indicator value, U (X_j_) is the membership function value of the jth comprehensive indicator, X_max_ and X_min_ are the maximum value and minimum value of the jth comprehensive indicator, respectively.

$$W_{j}=P_{j}/\sum_{j=1}^{m}P_{j}$$

where W_j_ is the ratio of the jth comprehensive indicator contribution to the total contribution rate of all comprehensive indicators and P_j_ is the contribution rate of the jth comprehensive indicator.

$$D=\sum_{j=1}^{m}\left[U\left(X_{j}\right)\times W_{j}\right]$$

where D is the comprehensive evaluation value for quality of rice varieties under different N treatments. 

## 3. Results

### 3.1. Trait Variations among Varieties and Nitrogen Levels

**Figure 1:**
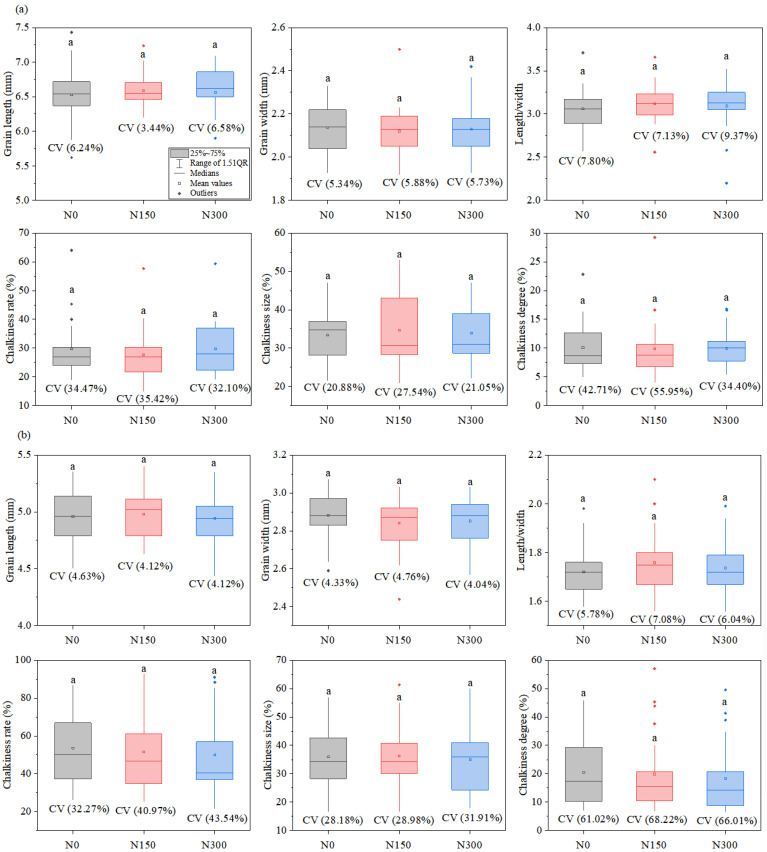
Distribution chart of grain shape and appearance quality for hybrid indica rice varieties (**a**) and inbred japonica rice varieties (**b**) with different nitrogen levels. The values of different lowercase letters refer to significant differences at *p* < 0.05 among different nitrogen levels. The CV value refers to the coefficient of variation. N0, N150, and N300 refer to treatments with 0 kg ha^−1^, 150 kg ha^−1^, and 300 kg ha^−1^ applied.

Significant interactions between varieties and nitrogen treatments for chalkiness traits were observed, the differences among hybrid indica rice varieties were the highest under the N150 nitrogen level. The ranges of chalkiness rate, chalkiness size, and chalkiness degree were 15.0–57.7%, 21.0–53.0%, and 4.1–29.2%, respectively; the coefficient of variation values were 35.4%, 27.5%, and 55.9%, respectively (Figure 1a). The highest differences among inbred japonica rice varieties were observed under the N300 nitrogen level. The ranges of chalkiness rate, chalkiness size, and chalkiness degree were 21.7.0–91.0%, 18.0–60.0%, and 6.7–49.5%, respectively; the coefficient of variation values were 43.5%, 31.9%, and 66.0%, respectively (Figure 1b). The differences among inbred japonica rice varieties were higher than those of the hybrid indica varieties. The differences of grain type among hybrid indica rice varieties were higher than those of the inbred japonica rice, and nitrogen fertilizer had little effect on grain type.

**Figure 2:**
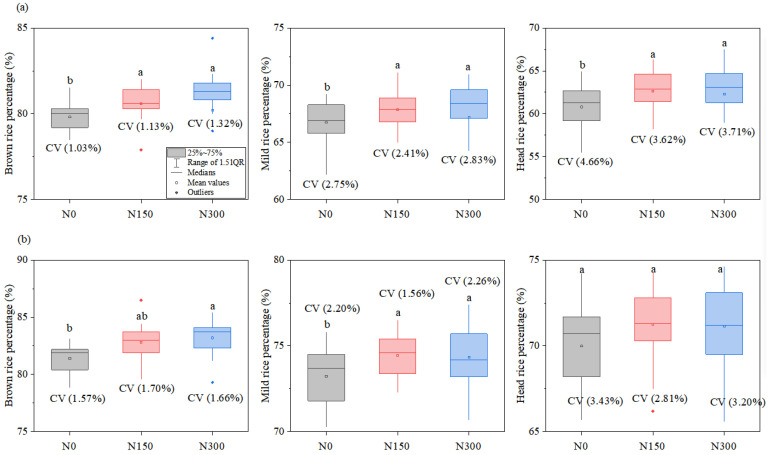
Distribution chart of milling quality for hybrid indica rice varieties (**a**) and inbred japonica rice varieties (**b**) with different nitrogen levels. The values of different lowercase letters refer to significant differences at *p* < 0.05 among different nitrogen levels. The CV value refers to the coefficient of variation. N0, N150, and N300 refer to treatments with 0 kg ha^−1^, 150 kg ha^−1^, and 300 kg ha^−1^ applied.

The differences of brown rice percentage among hybrid indica rice varieties were less than those of inbred japonica rice, while the differences in milled rice percentage and head rice percentage were converse. On the whole, with an increase in nitrogen levels from N0 to N150, the milling qualities significantly increased for both hybrid indica rice and inbred japonica rice, there was no significant difference between N150 and N300, and no significant interactions between varieties and nitrogen levels were observed for milling qualities (Figure 2).

**Figure 3:**
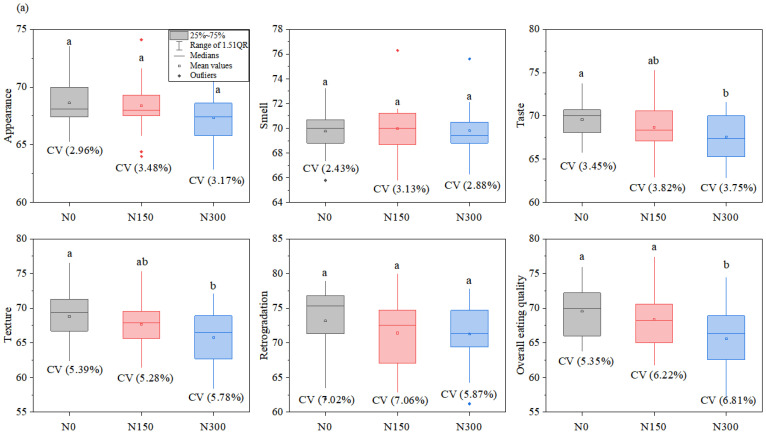

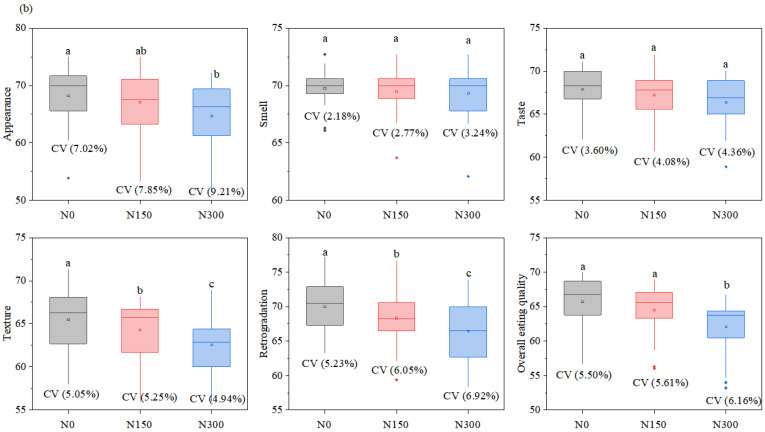
Distribution chart of eating quality for hybrid indica rice varieties (**a**) and inbred japonica rice varieties (**b**) with different nitrogen levels. The values of different lowercase letters refer to significant differences at *p* < 0.05 among different nitrogen levels. The CV value refers to the coefficient of variation. N0, N150, and N300 refer to treatments with 0 kg ha^−1^, 150 kg ha^−1^, and 300 kg ha^−1^ applied.

In terms of eating qualities, the differences of retrogradation among hybrid indica rice varieties were the highest, followed by overall eating quality, texture, taste, appearance, and smell. With an increase in nitrogen levels, the values of taste, texture, and overall eating quality significantly decreased, varieties × nitrogen level interactions were significant for texture and overall eating quality (Table 1 and Figure 3a). The differences of appearance among inbred japonica rice varieties were the highest, followed by retrogradation, overall eating quality, texture, taste, and smell. Nitrogen level had a significant effect on texture, retrogradation, and overall eating quality; significant interactions between varieties and nitrogen were also observed (Table 1 and Figure 3b).

### 3.2. Comprehensive Evaluation of Rice Quality of Different Varieties under Different Nitrogen Levels

**Figure 4:**
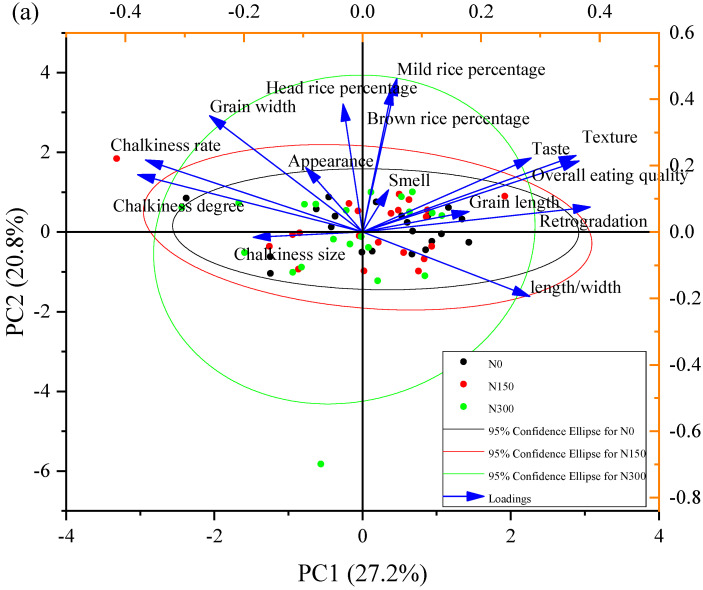

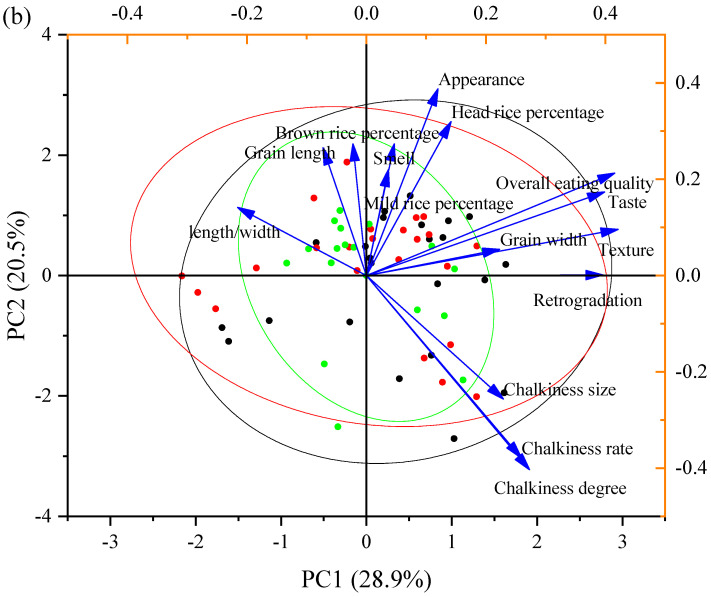
PCA distribution of hybrid indica rice varieties (**a**) and inbred japonica rice varieties (**b**) under three nitrogen levels. N0, N150, and N300 refer to treatments with 0 kg ha^−1^, 150 kg ha^−1^, and 300 kg ha^−1^ applied.

For hybrid indica rice, the PCA analysis showed that the KMO value was 0.62 and the significance level was 0.000, indicating that the principal component analysis could be performed. In the PCA analysis (Figure 4a and Table 2), the cumulative contributions to total variation of population from first to fifth principal components reached over 80.03%, which was enough to represent a large part of the information of the original indicators. The first principal component featured the largest contribution rate and eigenvalue, which were 27.21% and 4.08, respectively. PC1 was highly positively correlated with retrogradation, followed by overall eating quality and texture; chalkiness rate and chalkiness degree were negative values. The contribution rate and eigenvalue of PC2 were 20.76% and 3.11, respectively, which mainly reflected grain width, brown rice percentage, mild rice percentage, and the head rice percentage. The contribution rate and eigenvalue of PC3 were 14.25% and 2.14, respectively; head rice percentage, appearance, and taste corresponded to the highest loading matrix, but the head rice percentage was a negative value. The contribution rate and eigenvalue of PC4 were 11.08% and 1.66, respectively, which mainly included grain length and length/width. The contribution rate and eigenvalue of PC4 were 6.71% and 1.01, respectively, which mainly included chalkiness size and smell.

For inbred japonica rice, the PCA analysis showed that the KMO value was 0.58 and the significance level was 0.000. In the PCA analysis (Figure 4b and Table 3), the cumulative contributions to total variation of population from the first to the fifth principal component reached over 84.22%; their contribution rates were 28.92%, 20.51%, 16.53%, 11.79%, and 6.47%. The highest eigenvalue of PC1 was 4.34; PC1 was highly correlated with taste, texture, retrogradation, and overall eating quality. The eigenvalue of PC2 was 3.08, which was positively correlated with appearance and negatively correlated with chalkiness rate and chalkiness degree. The eigenvalue of PC3 was 2.48, which mainly reflected the milling qualities. The eigenvalue of PC4 was 1.77, and the length/width and chalkiness size corresponded to the highest loading matrix. The lowest eigenvalue of PC4 was 0.97, which mainly reflected grain length, grain width, and smell.

Based on the PCA results, the scores of the comprehensive indexes were obtained, and then were used in the subordinate function analysis (Tables S2 and S3). Using comprehensive index Z1 as an example, the maximum subordinate function value was 1.92 for N150-10, while the minimum value was −3.32 for N150-1. This suggested that, when only considering Z1, N150-10 showed the highest quality, while N150-1 had the weakest quality. Combined with the subordinate function values, μ (Xj), we calculated the index weight (Wj) of each comprehensive indicator of hybrid indica rice, which were 34.01%, 25.94%, 17.81%, 13.85, and 8.40% for the first, second, third, fourth, and fifth principal components, respectively; for inbred japonica rice, the index weight values were 34.34%, 24.35%, 19.62%, 14% and 7.68% for the first, second, third, fourth, and fifth principal components, respectively. Furthermore, the comprehensive evaluation (D) values of qualities of different rice varieties were calculated and ranked according to the formula given in the Material and Methods section. The D values of the top ranked varieties were higher, which indicated that they had a high comprehensive quality.

Taking the D value as a dependent variable and 15 quality traits as independent variables (Table S4), the optimal regression equation was constructed by stepwise regression analysis for hybrid indica rice, i.e., D = 0.004X15 + 0.067X1 + 0.010X12 + 0.007X11 + 0.061X3 + 0.005X10 + 0.006X13 − 2.241, and for inbred japonica rice, i.e., D = 0.014X6 + 0.011X15 + 0.021X5 + 0.001X7 + 0.140X1 + 0.013X4 + 0.005X14 − 0.183X3 − 4.535, where X1, X3, X4, X5, X6, X7, X10, X11, X12, X13, X14, and X15 refer to grain length, length/width, brown rice percentage, mild rice percentage, head rice percentage, chalkiness rate, appearance, smell, taste, texture, retrogradation, and overall eating quality, respectively. The determining coefficients of the two equations were both 0.99, that is, R^2^ = 0.99. The above results showed that the equations were reliable to evaluate the comprehensive quality of rice.

### 3.3. Cluster Analysis on the Quality of Different Rice Varieties

**Figure 5:**
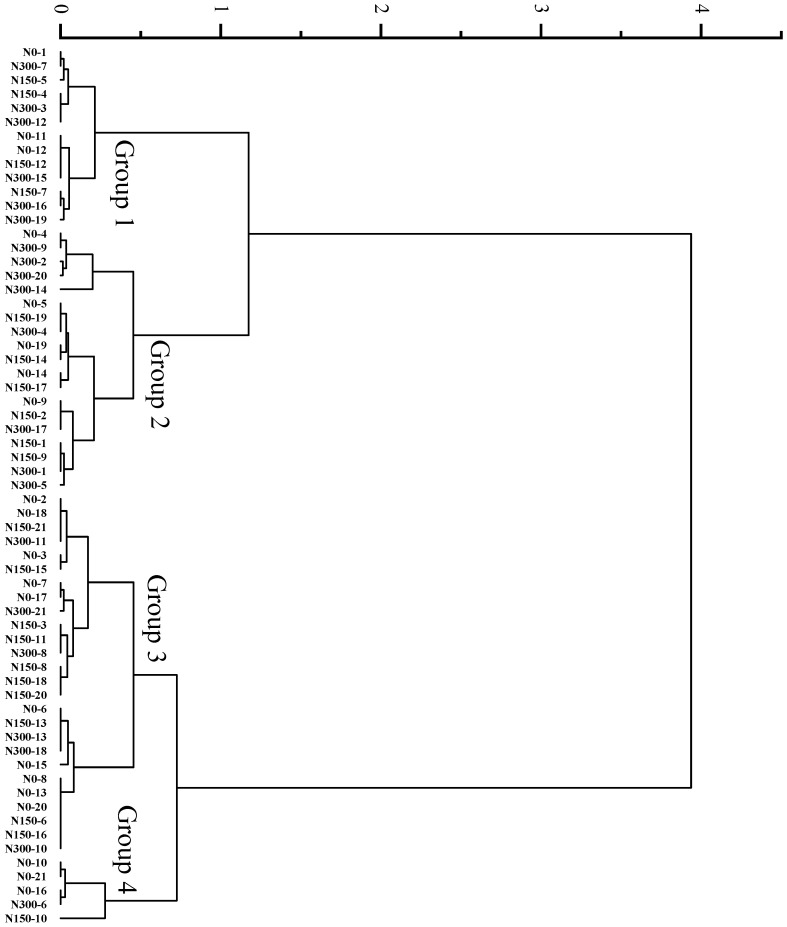
Cluster analysis of D values of various hybrid indica rice varieties under different nitrogen fertilizer conditions. Group 1 refers to the highest D value type, followed by Groups 2, 3, and 4.

**Figure 6:**
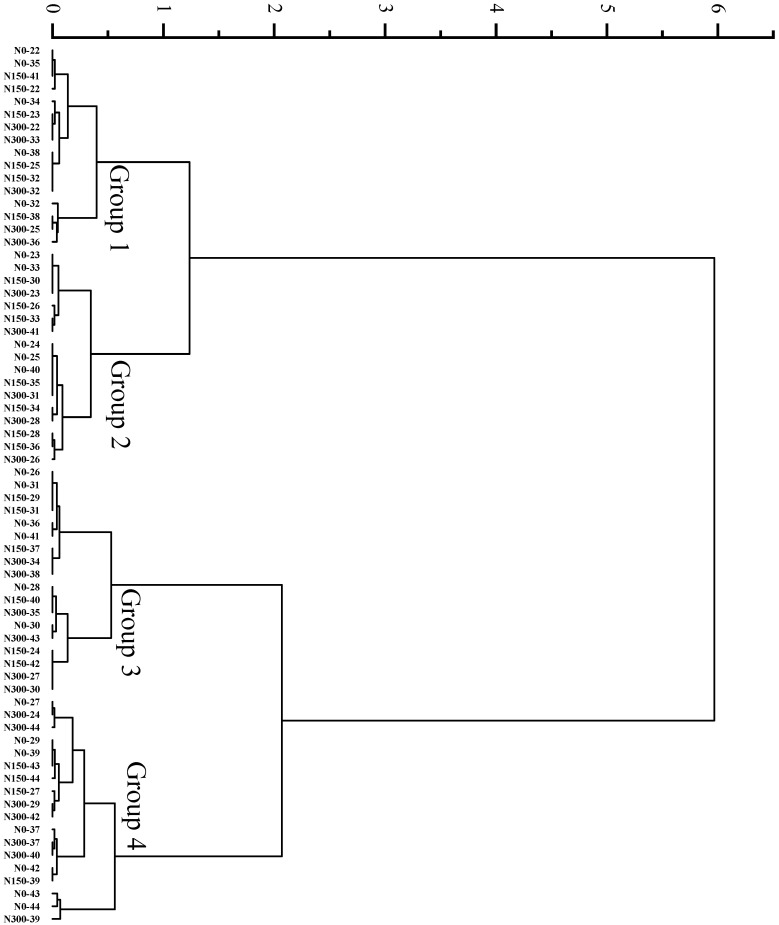
Cluster analysis of D values of various inbred japonica rice varieties under different nitrogen fertilizer conditions. Group 1 refers to the highest D value type, followed by Groups 2, 3, and 4.

Clustering analysis of D values assigned the 21 hybrid indica rice varieties under three nitrogen treatments into four major groups at a Euclidean distance of 0.5, the average D value of Group 1 was the highest, followed by Groups 2, 3, and 4, which were 0.76, 0.66, 0.59, and 0.51, respectively (Figure 5). Clustering analysis of D values assigned the 23 inbred japonica rice varieties under three nitrogen treatments into four major groups at a Euclidean distance of 1, the average D values of Groups 1, 2, 3, and 4 were 0.69, 0.61, 0.52, and 0.40, respectively (Figure 6).

**Figure 7:**
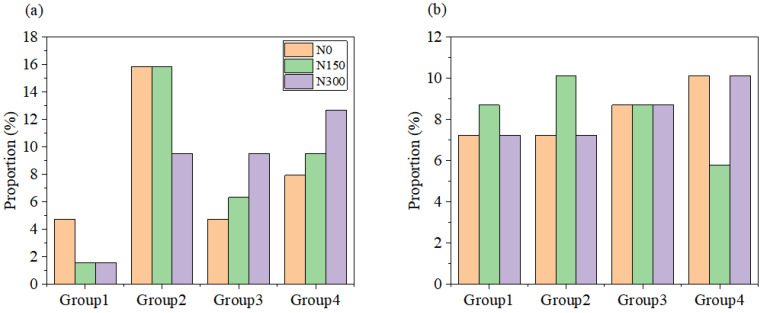
Proportion of rice samples in each group of hybrid indica rice (**a**) and inbred japonica rice (**b**) based on D value. Group 1 refers to the highest D value type, followed by Groups 2, 3, and 4.

For hybrid indica rice, the sum proportion of rice samples in the N0 subset of Groups 1 and 2 was higher than that in Groups 3 and 4, and the sum proportion of rice samples in the N150 or N300 subset of Groups 3 and 4 was higher than that in Groups 1 and 2 (Figure 7a). While for inbred japonica rice, the sum proportion of rice samples in the N150 subset of Groups 1 and 2 was higher than that in Groups 3 and 4, and the sum proportion of rice samples in the N0 or N300 subset of Groups 3 and 4 was higher than that in Groups 1 and 2 (Figure 7b).

## 4. Discussion

### 4.1. Difference in Rice Qualities among Varieties

In recent years, the breeding of high-quality rice in China has developed by leaps and bounds. In 2018, 268 rice varieties were approved by the state and the quantity of high-quality rice reached 50.0%, whereas 675 varieties were approved locally and the quantity of high-quality rice reached 24.6% [25]. Previous studies have shown that the chalkiness percentage was still the main quality index to limit the quality of rice in the early 21st century, and head rice percentage was an important index to limit the quality rate of rice at present [26,27,28]. Zeng et al. [29] considered that the future breeding of high-quality rice should focus on improving the appearance quality and milling quality of japonica rice, and the eating quality of indica rice. In the present study, we found that there were significant differences in appearance quality among varieties, especially inbred japonica rice, which indicated that the appearance quality of southern rice had improved significantly after 2000. However, milling quality had little change, and head rice rate of indica rice was basically less than 65%, while that of japonica rice was less than 75%, which may have been due to a change in grain shape, since slender rice is becoming more and more popular, but it is easy to break. Interestingly, it was also found that the difference in retrogradation among hybrid indica rice varieties was the largest, followed by taste, which may be related to the decrease of amylose content in indica hybrid rice in recent years [30]. However, a large difference in the appearance of cooked rice among inbred japonica rice varieties was found, followed by retrogradation, which may be related to the breeding of high-quality soft japonica rice varieties in recent years. Ma et al. [4] and Zhu et al. [31] found that the appearance of southern high-quality soft japonica rice with low amylose content was relatively turbid.

### 4.2. Nitrogen Effects on Rice Qualities

Nitrogen fertilizer is an important factor that affects rice quality. In this study, we found that properly increasing the amount of nitrogen application could improve the milling quality of hybrid indica rice and inbred japonica rice, which was consistent with the results of many studies. The reason for this improvment was that the increased nitrogen content in the plant promoted the transport of nutrients from various parts to the panicle and increased grain hardness, thus, enhancing the milling resistance of rice and significantly improving the percentage of head rice [32]. However, due to the differences in variety types and environmental conditions, research results may differ. Zhou et al. [33] found that the response of head rice percentage to nitrogen fertilizer was different under different temperature and light conditions. Meng et al. [34] discovered that the application of N fertilizer deteriorated milling quality of modern cultivars of inbred japonica rice.

Many scholars have reported different research results on the effect of nitrogen application rate on the appearance quality of rice. The study of double-cropping late indica rice by Tang et al. [35] showed that proper application of nitrogen fertilizer could improve the appearance quality of rice. The reason was that proper application of nitrogen fertilizer could prolong the period of grain filling, slow down the grain filling rate, and provide material guarantee for grain filling, thus, reducing the formation of rice chalkiness. Tao et al. [36] found that the chalkiness degree of modern middle indica rice varieties significantly increased after the application of nitrogen fertilizer. Zhang et al. [37] found that with an increase in nitrogen level, the chalkiness rate and chalkiness degree decreased at first and then increased. In this study, we found that the different effects of nitrogen on appearance quality varied with varieties. For future studies, it is necessary to analyze the mechanism of nitrogen effect on rice chalkiness formation from the interaction of carbon and nitrogen metabolism.

In this study, we found that, on the whole, the eating quality decreased with the application of nitrogen fertilizer, but there were significant differences in the responses of different varieties to nitrogen fertilizer. The texture of hybrid indica rice varied greatly in response to nitrogen fertilizer, and the texture and retrogradation of inbred indica rice varied greatly in response to nitrogen fertilizer. This was similar to the results of previous studies. A study on Nanjing series soft rice by Zhu et al. [38] showed that the taste value of rice decreased in stages, that is, in the range of 0–262.5 kg ha^−1^ nitrogen application, the taste value decreased slowly, and decreased rapidly when the nitrogen application rate was more than 300 kg ha^−1^. The decreased rice viscosity, palatability, and retrogradation degree of japonica rice under high nitrogen may be caused by an increase in grain protein content and the change in fine structure of amylopectin [39]. However, some other studies have found that the appropriate amount of nitrogen application could improve the cooking and eating quality of rice by prolonging the filling time of inferior grains [40]. Optimization of nitrogen fertilization could decrease the starch large granule; increase the α-1,6 linkage, amylopectin long chains, and relative crystallinity [41]; increase the proportion of amylopectin short branch chains, and the viscosity of starch paste; decrease the ratio of amylose to amylopectin and starch gelatinization temperature [42].

Related high-quality cultivation techniques have been applied to production practice, but there are still many problems: (1) Rice quality is affected by genetics, therefore, the nitrogen uptake characteristics of different varieties and types of rice are different, and relatively reasonable nitrogen fertilizer level and operation methods should be put forward for varieties with different nitrogen responses in the future. (2) The effect of nitrogen fertilizer on rice quality may be different due to different conditions such as temperature, light, and soil fertility.

### 4.3. Comprehensive Evaluation of Rice Quality

At present, an objective, simple and fast evaluation system which is established by using the membership function method and principal component analysis in mathematics can reflect the comprehensive quality of rice. For example, Shi et al. [43] evaluated 17 quality traits by using 7 rice varieties with 12 nitrogen treatments, and established a comprehensive evaluation equation of rice quality in Hubei Province. Xu et al. [21] established a prediction model of the relationships among rice quality and geographical location, soil basic fertility, and nitrogen application level, through experiments at different ecological sites in southwest China. However, in the above-mentioned studies, only a few varieties were selected, and therefore, the results may not be representative. In this study, forty-four rice varieties widely planted in South China were performed at different nitrogen levels, and through principal component analysis, membership function method and stepwise regression analysis, the comprehensive quality evaluation equations of hybrid indica rice and inbred japonica rice were established. It was found that, in general, the nitrogen application rate of hybrid indica rice could be appropriately reduced, and that of inbred japonica rice could be increased properly, which could improve the comprehensive quality of rice. However, the specific measures should be adjusted according to different varieties, which would provide a theoretical basis for the breeding of high-quality varieties and high-quality cultivation techniques in the middle and lower reaches of the Yangtze River.

In the future, the qualities of different types of rice (main varieties in different locations) should be investigated under multi-environments (geographical environment, light and temperature environment, and cultivation measures) to establish a comprehensive prediction model of the relationships among the quality of different rice types and geographical location, soil basic fertility, cultivation measures, and light-temperature environment.

## 5. Conclusions

Nitrogen treatments and rice varieties have complex effects on rice qualities. Among the fifteen quality traits, the variation of chalkiness traits was the largest. The comprehensive quality differences among inbred japonica varieties were mainly due to the differences in head rice percentages. The comprehensive quality differences among inbred japonica varieties were mainly due to the differences in eating values by sensory evaluation. It was found that, for hybrid indica rice, the amount of nitrogen application could be reduced appropriately, and for japonica rice, the amount of nitrogen application could be increased appropriately, to improve the comprehensive quality of rice.

## Figures and Tables

**Table 1:** Significance of variance estimates related to varieties, nitrogen levels, and their interactions on grain qualities.

Quality Traits		Hybrid Indica Rice			Inbred Japonica Rice	
	V	N	V × N	V	N	V × N
Grain length (mm)	**	ns	ns	**	ns	ns
Grain width (mm)	**	ns	ns	**	ns	ns
Length/width	**	ns	ns	**	ns	ns
Brown rice percentage (%)	*	**	ns	*	**	ns
Mild rice percentage (%)	*	*	ns	*	*	ns
Head rice percentage (%)	*	**	ns	**	ns	ns
Chalkiness rate (%)	**	ns	**	**	ns	**
Chalkiness size (%)	**	ns	*	**	ns	ns
Chalkiness degree (%)	**	ns	**	**	ns	ns
Appearance	*	ns	*	**	ns	ns
Aroma	**	ns	ns	**	ns	ns
Taste	**	*	ns	**	ns	ns
Texture	**	*	**	**	*	*
Retrogradation	**	ns	ns	**	*	**
Overall eating quality	**	*	*	**	**	*

ns, not significant; * and **, significance at *p* < 0.05 and *p* < 0.01, respectively; V, varieties; N, nitrogen levels.

**Table 2:** Eigenvalue and contribution of each comprehensive index and loading matrix of each component for qualities of hybrid indica rice.

Traits	PC1	PC2	PC3	PC4	PC5
Grain length	0.1795	0.0618	0.0384	0.6309	0.2444
Grain width	−0.2582	0.3504	0.2056	−0.1808	0.0972
length/width	0.2821	−0.1943	−0.1126	0.5546	0.0892
Brown rice percentage	0.0502	0.4340	−0.3094	0.1385	−0.1573
Mild rice percentage	0.0577	0.4621	−0.3377	0.0508	−0.0509
Head rice percentage	−0.0328	0.3856	−0.4229	0.0275	−0.0704
Chalkiness rate	−0.3661	0.2167	0.1745	0.1119	0.1687
Chalkiness size	−0.1842	−0.0158	0.1995	0.2449	−0.4473
Chalkiness degree	−0.3794	0.1728	0.2446	0.2258	−0.0943
Appearance	−0.0958	0.1943	0.3518	0.2578	0.0145
Smell	0.0438	0.1270	−0.0018	−0.1190	0.7708
Taste	0.2845	0.2229	0.3686	−0.0470	0.0474
Texture	0.3602	0.2306	0.2861	−0.1046	−0.1015
Retrogradation	0.3842	0.0751	0.0357	−0.1381	−0.2071
Overall eating quality	0.3652	0.2138	0.2866	−0.0536	−0.0590
Eigenvalue	4.0828	3.1141	2.1377	1.6626	1.0080
Contributive ratio (%)	27.2187	20.7605	14.2513	11.0836	6.7199
Cumulative (%)	27.2187	47.9792	62.2305	73.3142	80.0341

**Table 3:** Eigenvalue and contribution of each comprehensive index and loading matrix of each component for qualities of inbred japonica rice.

Traits	PC1	PC2	PC3	PC4	PC5
Grain length	−0.0728	0.2654	−0.1763	0.3058	0.7037
Grain width	0.2231	0.0543	0.2978	−0.3500	0.4141
length/width	−0.2157	0.1415	−0.3356	0.4709	0.1813
Brown rice percentage	−0.0227	0.2734	0.4554	0.1874	−0.1644
Mild rice percentage	0.0367	0.2200	0.4433	0.3671	−0.0468
Head rice percentage	0.1417	0.3196	0.3985	0.1414	−0.0032
Chalkiness rate	0.2593	−0.3786	0.0400	0.1637	0.0868
Chalkiness size	0.2292	−0.2566	0.0237	0.4174	−0.1488
Chalkiness degree	0.2731	−0.4029	0.0358	0.2701	−0.0315
Appearance	0.1197	0.3878	−0.2309	−0.1766	−0.2567
Smell	0.0465	0.2735	−0.2926	0.2033	−0.3801
Taste	0.3985	0.1737	−0.1562	0.0309	−0.0616
Texture	0.4212	0.0960	−0.0886	−0.1316	0.1545
Retrogradation	0.3958	0.0011	−0.1002	0.0788	0.0485
Overall eating quality	0.4157	0.2127	−0.1424	−0.0376	0.0126
Eigenvalue	4.3386	3.0768	2.4791	1.7693	0.9703
Contributive ratio (%)	28.9239	20.5122	16.5271	11.7952	6.4686
Cumulative (%)	28.9239	49.4361	65.9632	77.7584	84.2270

## Data Availability

Data is contained within the article.

## Supplementary Materials

The following supporting information can be downloaded at: https://www.mdpi.com/article/10.3390/foods12040697/s1, Table S1: The instruction of rice varieties in this article; Table S2: Value of each comprehensive indicators (Z), subordinate function values, μ(X), comprehensive evaluation value (D), and order for hybrid indica rice varieties; Table S3: Value of each comprehensive indicators (Z), subordinate function values, μ(X), comprehensive evaluation value (D), and order for inbred japonica rice varieties; Table S4: Multiple regression analysis for determining correlations between comprehensive quality and 15 quality traits.

## References

[1] (2021). National Bureau of Statistics of the People’s Republic of China. China Statistical Yearbook.

[2] Li X.K., Wu L., Geng X., Xia X.H., Wang X.H., Xu Z.J., Xu Q. (2018). Deciphering the environmental impacts on rice quality for different rice cultivated areas. Rice.

[3] Cui Y., Zhu M.M., Xu Z.J., Chen W.F. (2020). The breeding of japonica rice in northern China: An eleven-year study (2006–2016). Journal of Integrative Agriculture.

[4] Ma H.Z., Chen X.Y., Wang Z.J., Zhu Y., Jiang W.Q., Ren G.L., Ma Z.T., Wei H.Y., Zhang H.C., Liu G.D. (2021). Analysis on appearance and cooking taste quality characteristics of some high quality japonica rice in China. Sci. Agric. Sin..

[5] Wang C.L., Zhang Y.D., Zhu Z., Chen T., Zhao Q.Y., Zhong W.G., Yang J., Yao S., Zhou L.H., Zhao L. (2017). Research progress on the breeding of japonica super rice varieties in Jiangsu Province, China. J. Integr. Agric..

[6] Cheng F., Xu Q., Xu Z.J., Chen W.F. (2020). Effect of rice breeding process on improvement of yield and quality in china. Rice Sci..

[7] (2017). High Quality Paddy. Standardization Administration of the People’s Republic of China.

[8] Zhang Y.D., Chen T., Zhao Q.Y., Zhu Z., Zhou L.H., Yao S., Zhao L., Wang C.L. (2011). Establishment and application of evaluation method for eating quality of rice. Jiangsu Agric. Sci..

[9] Meng Q.H., Li X.H., Sanshang L.S., Heye Y.X., Lu S.W., Chen A.H., Yao X.M., Guan H.T. (2010). Predicting eating quality of short grain rice by using visible and near infrared spectroscopy. J. Chin. Creals Oils Assoc..

[10] Xu Y.J., Ying Y.N., Ouyang S.H., Duan X.L., Sun H., Jiang S.K., Sun S.C., Bao J.S. (2018). Factors Affecting Sensory Quality of Cooked japonica Rice. Rice Sci..

[11] Zhao C.F., Yue H.L., Huang S.J., Zhou L.H., Zhao L., Chen T., Zhu Z., Zhao Q.Y., Yao S., Liang W.H. (2019). Eating quality and physicochemical properties in nanjing rice varieties. Sci. Agric. Sin..

[12] Huang M., Hu L.Q., Cao J.L., Zhang R.C., Chen J.N., Cao F.B., Liu L.S., Fang S.L., Zhang M. (2022). Texture and digestion properties of hybrid rice: A comparison between two cultivars released 18 years apart. Food Chem. X.

[13] Chen N., Yang S.H., Xie L.H., Daun B.W. (2011). Differences in sensory evaluation of cooked rice in indica rice growing region in China. China Jounal Rice Sci..

[14] Lu H., Yuan Y.J., Zhang S.Q., Chen H., Chen D., Zhong X.Y., Li B., Deng F., Chen Y., Li G.Y. (2021). Evaluation of rice eating quality and optimization of varieties of Southwest indica hybrid rice based on three taste evaluation methods. Sci. Agric. Sin..

[15] Chen H., Chen D., He L.H., Wang T., Lu H., Yang F., Deng F., Chen Y., Tao Y.F., Li M. (2021). Correlation of taste values with chemical compositions and Rapid Visco Analyser profiles of 36 indica rice (*Oryza sativa* L.) varieties. Food Chem..

[16] Zhu Z., Xiong N., Liu H., Wu L., Meng H., Liu L., Wu L.L. (2016). Establishment of models to evaluate the eating quality and comprehensive quality of indica rice. Food Sci..

[17] Jiang X.J., Zhou J.M., Huang M., Duan G.J., Luo H.G., Ou L.J., Ni X.L. (2009). Evaluation of rice quality of new rice varieties in Hunan Province based on principal component and cluster Analysis. China Rice.

[18] Jing Z.Y., Wei J.Q., Wang L.Y., Song W.M., Zheng J.P., Guo Y.X. (2020). Comprehensive quality evaluation of different rice varieties based on principal component analysis. Food Sci..

[19] Chen Y.Y., Hu X.X., Chen J.D., Yang X., Ma Q., Chen Q., Ge M.J., Dai Q.G. (2012). Effect of Nitrogen Fertilizer Application on Eating Quality of Early-Maturing Late Japonica Rice in Jiangsu and Its Difference among Varieties. Acta Agron. Sin..

[20] Liao S., Deng F., Tian Q.L., Li W., Hu H., Pu S.L., Li S.X., Ren W.J. (2018). Response of major restore lines for hybrid rice to nitrogen rate in Sichuan Province. J. Plant Nutr. Fertil..

[21] Xu F.X., Xiong H., Zhang L., Guo X.Y., Zhu Y.C., Zhou X.B., Liu M. (2012). Effect factor and predict model of rice quality variation for mid-season hybrid rice at different ecological sites and nitrogen application levels. Chin. J. Rice Sci..

[22] Wei H.Y., Zhu Y., Qiu S., Han C., Hu L., Xu D., Zhou N.B., Xing Z.P., Hu Y.J., Cui P.Y. (2018). Combined effect of shading time and nitrogenlevel on grain filling and grain quality in japonica super rice. J. Integr. Agric..

[23] Yang X.Y., Lin Z.M., Liu Z.H., Alim M.A., Bi J.G., Li G.H., Wang Q.S., Wang S.H., Ding Y.F. (2013). Physicochemical and sensory properties of japonica rice varied with production areas in china. J. Integr. Agric..

[24] Yao D.P., Wu J., Luo Q.H., Shen H., Zhuang W., Xiao G., Li J.W., Li Y.G., Deng Q.Y., Lei D.Y. (2021). Comprehensive evaluation of high temperature tolerance of six rice varieties during grain-filling period based on key starch physicochemical indexs. LWT-Food Sci. Technol..

[25] Lv F., Yang F., Fan T., Liu J., Li Q., Wang L.G., Long X.B. (2019). Analysis of rice variety approval data from 1977 to 2018. Chin. Seed Ind..

[26] Zhang H., Tan G.L., Sun X.L., Liu L.J., Yang J.C. (2009). Changes in grain quality during the evolution of mid-season indica rice cultivars in Jiangsu province. Acta Agron. Sin..

[27] Gu J.F., Chen J., Chen L., Wang Z.Q., Zhang H., Yang J.C. (2015). Grain quality changes and responses to nitrogen fertilizer of japonica rice cultivars released in the Yangtze River Basin from the 1950s to 2000s. Crop J..

[28] Hu X.Q., Zhang W.X., Shao Y.F., Yu Y.H., Lu L., Chen M.X. (2021). Analysis on high quality rate of rice in China during recent 20 years. China Rice.

[29] Zeng Y.H., Tan X.M., Zeng Y.J., Xie X.B., Pan X.H., Shi Q.H., Zhang J. (2019). Changes in the rice grain quality of different high-quality rice varieties released in southern China from 2007 to 2017. J. Cereal Sci..

[30] Feng F., Li Y.J., Qin X.L., Liao Y.C., Siddique K.H.M. (2017). Changes in rice grain quality of indica and japonica type varieties released in China from 2000 to 2014. Front. Plant Sci..

[31] Zhu D.W., Zhang L.P., Chen M.X., Fang C.Y., Yu Y.H., Zheng X.L., Shao Y.F. (2022). Characteristics of high-quality rice varieties and taste sensory evaluation values in China. Sci. Agric. Sin..

[32] Ma Q., Zhang H.C., Dai Q.G., Wei H.Y., Huo Z.Y., Xu K., Yin C.Y., Hang J., Zhang S.F., Zhang Q. (2009). Effects of Nitrogen Application Rate and Growth-Development Type on Milling Quality in Japonica Rice. Acta Agron. Sin..

[33] Zhou L.J., Liang S.S., Ponce K., Marundon S., Ye G.Y., Zhao X.Q. (2015). Factors affecting head rice yield and chalkiness in indica rice. Field Crops Res..

[34] Meng T.Y., Zhang X.B., Chen X., Ge J.L., Zhou G.S., Wei H.H., Dai Q.G. (2022). Trends in grain quality and responses to nitrogen application of japonica inbred rice released after the 1980s in east China. Cereal Chem..

[35] Tang J., Tang C., Guo B.W., Zhang C.X., Zhang Z.Z., Wang K., Zhang H.C., Chen H., Sun M.Z. (2020). Effect of nitrogen application on yield and rice quality of mechanical transplanting high quality late rice. Acta Agron. Sin..

[36] Tao J., Qian X.C., Jv C.X., Liu L.J., Zhang H., Gu J.F., Wang Z.Q., Yang J.C. (2016). Grain quality and its response to nitrogen fertilizer in mid-season indica rice varieties planted in different decades from 1950s to 2010s. Acta Agron. Sin..

[37] Zhang Q., Guo B.W., Hu Y.J., Zhang H.C., Xu Y.F., Xu X.J., Zhu B.H., Xu J.F., Niu Z.Y., Tu R.W. (2021). Differences in yield and rice quality of soft japonica rice with high quality and high yield under different nitrogen levels. Chin. J. Rice Sci..

[38] Zhu D.W., Zhang H.C., Guo B.W., Xu K., Dai Q.G., Wei H.Y., Gao H., Hu Y.J., Cui P.Y., Huo Z.Y. (2017). Effects of nitrogen level on yield and quality of japonica soft super rice. J. Integr. Agric..

[39] Huang S.J., Zhao C.F., Zhu Z., Zhou L.H., Zheng Q.H., Wang C.L. (2020). Characterization of eating quality and starch properties of two Wx alleles japonica rice cultivars under different nitrogen treatments. J. Integr. Agric..

[40] Zhang J., Zhang Y.Y., Song N.Y., Chen Q.L., Sun H.Z., Peng T., Huang S., Zhao Q.Z. (2021). Response of grain-filling rate and grain quality of mid-season indica rice to nitrogen application. J. Integr. Agric..

[41] Xiong R.Y., Tan X.M., Yang T.T., Pan X.H., Zeng Y.J., Huang S., Shang Q.Y., Zhang J., Zeng Y.H. (2022). Relation of cooked rice texture to starch structure and physicochemical properties under different nitrogen managements. Carbohydr. Polym..

[42] Zhou T.Y., Zhou Q., Li E.P., Yuan L.M., Wang W.L., Zhang H., Liu L.J., Wang Z.Q., Yang J.C., Gu J.F. (2020). Effects of nitrogen fertilizer on structure and physicochemical properties of ‘super’ rice starch. Carbohydr. Polym..

[43] Shi S.J., Wang E.T., Li C.X., Zhou H., Cai M.L., Cao C.G., Jiang Y. (2021). Comprehensive evaluation of 17 qualities of 84 types of rice based on principal component analysis. Foods.

