# Supplementary material for: Comprehensive Evaluation of Rice Qualities under Different Nitrogen Levels in South China
Source: Foods. 2023 Feb 6;12(4):697. doi: 10.3390/foods12040697 (PMC9956055; doi:10.3390/foods12040697)
Supplement: Supplementary file 1 [file foods-12-00697-s001.zip › foods-2122722-supplementary.pdf]

Table S1. The instruction of rice varieties in this article.

Rice type	Serial number	Variety	Year of official release	Heading date	Maturity date
Hybrid indica rice	1	Shanyou63	1981	8/31	10/12
	2	Liangyoupeijiu	1999	9/2	10/12
	3	Xinliangyou6	2003	8/21	9/25
	4	Shengliangyou5814	2007	9/2	10/12
	5	Tianyouhuazhan	2007	8/24	9/25
	6	Zhongzheyou1	2001	9/2	10/12
	7	Cliangyouhuazhan	2010	8/26	10/8
	8	Yliangyou900	2013	9/3	10/12
	9	Chaoyou1000	2014	8/30	10/12
	10	Fengyouxiangzhan	2001	8/30	10/8
	11	Huiliangyou898	2011	8/27	10/8
	12	Quanliangyou3301	2013	9/1	10/12
	13	Zhongzheyou8	2001	9/3	10/9
	14	Qianyou930	2009	9/2	10/8
	15	Guangliangyou66	2005	9/2	10/8
	16	Jvliangyou60	2012	8/28	10/5
	17	Yliangyou1	2005	9/2	10/9
	18	Yangliangyou6	2009	9/2	10/9
	19	Liangyou688	2008	9/3	10/9
	20	Pengyou6377	2016	8/23	10/8
	21	Zhaoyou5431	2016	9/6	10/20
Inbred japonica rice	22	Ningjing1	2001	9/2	10/23
	23	Ningjing3	2001	9/2	10/23
	24	Ningjing7	2011	8/30	10/27
	25	Ningjing8	2012	8/31	10/29
	26	Wuyunjing23	2005	9/2	10/27
	27	Wuyunjing24	2006	8/27	10/27
	28	Nanjing9108	2009	8/27	10/27
	29	Nanjing5055	2005	9/2	10/29
	30	Nanjing0212	2010	8/27	10/27
	31	Nanjing52	2009	8/28	10/23
	32	Nanjing46	2004	9/9	11/4
	33	Wuyujing3	1990	8/30	10/23
	34	Wujing14	1999	9/2	10/24
	35	Wuyunjing31	2010	9/2	10/24
	36	Ningjing2	2001	9/1	10/24
	37	Suxiangjing3	2006	8/18	9/25
	38	Suxiangjing100	2011	9/9	11/4
	39	Nongken57	1957	8/27	9/25
	40	Wuyunjing7	1995	8/31	11/1

	41	Huaidao5	1994	8/29	10/25
	42	Ningjing4	2007	8/25	10/24
	43	Ningjing5	2007	8/27	10/18
	44	Ningjing6	2009	8/25	10/13

Table S2. Value of each comprehensive indicators (Z), subordinate function values, μ(X), comprehensive evaluation value (D) and order for hybrid indica rice varieties.

Type	Z1	Z2	Z3	Z4	Z5	U1	U2	U3	U4	U5	D	Order	Cluster
N0-1	-2.38	0.85	1.16	0.99	1.50	0.18	0.87	0.63	0.82	0.74	0.57	22	1
N0-11	0.67	-0.55	-0.33	0.75	-1.70	0.76	0.69	0.35	0.78	0.06	0.61	31	1
N0-12	-0.42	0.13	0.93	-0.26	-0.03	0.55	0.78	0.58	0.60	0.41	0.61	30	1
N150-4	0.75	-0.98	-1.00	-0.50	0.14	0.78	0.63	0.22	0.55	0.45	0.58	24	1
N150-5	-0.86	-0.93	0.66	1.32	-1.21	0.47	0.64	0.53	0.88	0.16	0.56	20	1
N150-7	0.21	-0.26	-0.14	0.33	-0.80	0.67	0.73	0.38	0.70	0.25	0.60	28	1
N150-12	-0.06	0.53	-0.19	-0.26	0.09	0.62	0.83	0.37	0.60	0.44	0.61	32	1
N300-3	-0.02	-0.11	-1.57	0.28	1.40	0.63	0.74	0.11	0.70	0.71	0.58	25	1
N300-7	-0.17	-0.30	-0.48	0.83	-1.14	0.60	0.72	0.32	0.80	0.18	0.57	21	1
N300-12	-0.63	0.70	-0.43	0.97	-1.21	0.51	0.85	0.33	0.82	0.16	0.58	23	1
N300-15	-0.78	0.70	0.40	0.96	-0.66	0.48	0.85	0.48	0.82	0.28	0.61	29	1
N300-16	-0.22	0.54	-0.31	-0.21	0.23	0.59	0.83	0.35	0.61	0.46	0.60	27	1
N300-19	0.20	-1.22	-0.98	0.70	1.29	0.67	0.60	0.22	0.77	0.69	0.59	26	1
N0-4	0.13	-0.48	-1.51	-2.38	-0.62	0.66	0.70	0.12	0.22	0.28	0.48	4	2
N0-5	-1.24	-1.04	0.79	0.81	-0.15	0.40	0.62	0.56	0.79	0.39	0.54	16	2
N0-9	-1.24	-0.62	0.35	-0.33	-0.88	0.40	0.68	0.47	0.59	0.23	0.50	6	2
N0-14	-0.37	0.40	0.90	-1.86	-1.64	0.56	0.81	0.58	0.31	0.07	0.55	19	2
N0-19	-0.01	-0.50	-1.45	-0.97	0.88	0.63	0.69	0.13	0.47	0.60	0.53	14	2
N150-1	-3.32	1.84	1.42	0.78	-0.06	0.00	1.00	0.68	0.79	0.40	0.52	11	2
N150-2	-1.26	-0.36	-1.33	0.63	0.64	0.39	0.71	0.16	0.76	0.55	0.50	7	2
N150-9	-0.85	-0.02	-0.56	-0.03	-0.72	0.47	0.76	0.30	0.64	0.27	0.52	10	2
N150-14	-0.94	-0.06	0.29	-0.19	-1.30	0.45	0.75	0.46	0.61	0.14	0.53	13	2
N150-17	-0.04	-0.09	-1.51	-0.44	0.52	0.62	0.75	0.12	0.56	0.53	0.55	18	2
N150-19	0.02	-0.98	-1.18	-0.26	0.39	0.64	0.63	0.19	0.60	0.50	0.54	17	2
N300-1	-2.44	0.62	0.98	-0.77	2.06	0.17	0.84	0.59	0.51	0.85	0.52	12	2
N300-2	-1.59	-0.51	-1.55	0.54	0.33	0.33	0.69	0.11	0.74	0.49	0.46	2	2
N300-4	0.08	-0.38	-0.79	-1.75	0.22	0.65	0.71	0.26	0.33	0.46	0.54	15	2
N300-5	-0.82	-0.88	-1.12	1.72	-1.01	0.48	0.64	0.20	0.96	0.20	0.51	9	2
N300-9	-0.94	-1.01	-1.30	0.26	0.37	0.45	0.63	0.16	0.69	0.50	0.48	5	2
N300-14	-1.67	0.73	-0.89	-3.57	-1.74	0.31	0.85	0.24	0.00	0.05	0.38	1	2
N300-17	-0.39	-0.18	-2.16	0.15	-0.50	0.56	0.74	0.00	0.67	0.31	0.50	8	2
N300-20	-0.56	-5.82	3.13	-1.28	1.30	0.53	0.00	1.00	0.41	0.69	0.47	3	2
N0-2	-0.46	0.88	1.01	0.63	0.24	0.55	0.87	0.60	0.76	0.47	0.66	45	3
N0-3	0.53	0.41	0.24	-0.86	1.47	0.73	0.81	0.45	0.49	0.73	0.67	47	3
N0-6	0.94	-0.23	0.92	-0.33	0.25	0.81	0.73	0.58	0.58	0.47	0.69	55	3

N0-7	0.68	0.02	-0.29	-0.29	-0.12	0.76	0.76	0.35	0.59	0.39	0.63	35	3
N0-8	0.88	0.41	0.41	-0.35	-0.10	0.80	0.81	0.49	0.58	0.40	0.68	52	3
N0-13	1.07	-0.05	0.85	-0.35	-0.70	0.84	0.75	0.57	0.58	0.27	0.68	53	3
N0-15	1.16	0.61	1.42	-1.62	-0.37	0.85	0.84	0.68	0.35	0.34	0.71	58	3
N0-17	-0.62	0.58	1.30	0.14	-0.03	0.51	0.83	0.65	0.67	0.41	0.63	34	3
N0-18	0.18	0.75	0.50	-0.47	0.21	0.67	0.86	0.50	0.56	0.46	0.66	42	3
N0-20	0.85	-0.45	0.48	0.47	-0.14	0.80	0.70	0.50	0.73	0.39	0.68	48	3
N150-3	0.39	0.47	-0.69	-0.14	0.89	0.71	0.82	0.28	0.62	0.61	0.64	38	3
N150-6	0.63	0.81	-0.92	-0.56	2.75	0.75	0.87	0.23	0.54	1.00	0.68	51	3
N150-8	0.88	0.55	0.04	-1.21	-0.09	0.80	0.83	0.42	0.43	0.40	0.65	41	3
N150-11	0.93	-0.36	0.08	0.13	-1.59	0.81	0.71	0.42	0.67	0.08	0.64	36	3
N150-13	0.86	0.38	0.48	0.26	-0.45	0.80	0.81	0.50	0.69	0.32	0.69	57	3
N150-15	0.49	0.95	-0.05	-0.49	0.47	0.73	0.88	0.40	0.56	0.52	0.67	46	3
N150-16	0.48	0.55	0.65	0.07	-0.19	0.73	0.83	0.53	0.66	0.38	0.68	50	3
N150-18	-0.18	0.72	0.80	0.05	0.01	0.60	0.85	0.56	0.65	0.42	0.65	39	3
N150-20	0.56	-0.51	0.36	1.11	-0.97	0.74	0.69	0.48	0.85	0.21	0.65	40	3
N150-21	0.83	-0.67	-0.80	1.96	-0.04	0.79	0.67	0.26	1.00	0.41	0.66	44	3
N300-8	0.63	0.50	0.06	-1.19	-0.14	0.75	0.83	0.42	0.43	0.39	0.64	37	3
N300-10	0.94	0.48	-0.24	-0.59	0.68	0.81	0.82	0.36	0.54	0.56	0.68	49	3
N300-11	1.07	0.41	-0.23	0.31	-1.97	0.84	0.81	0.36	0.70	0.00	0.66	43	3
N300-13	0.53	0.89	0.30	1.08	-1.25	0.73	0.87	0.46	0.84	0.15	0.69	54	3
N300-18	0.11	1.00	0.26	0.59	0.90	0.66	0.89	0.46	0.75	0.61	0.69	56	3
N300-21	0.84	-1.10	-0.89	0.93	0.06	0.79	0.62	0.24	0.81	0.43	0.62	33	3
N0-10	1.34	0.33	1.37	-0.30	-0.20	0.89	0.80	0.67	0.59	0.37	0.74	61	4
N0-16	0.60	0.24	1.04	1.15	0.29	0.75	0.79	0.61	0.85	0.48	0.73	59	4
N0-21	1.43	-0.26	-0.22	1.85	0.57	0.91	0.73	0.37	0.98	0.54	0.74	62	4
N150-10	1.92	0.90	1.86	0.50	1.17	1.00	0.88	0.76	0.74	0.66	0.86	63	4
N300-6	0.67	1.01	-0.38	0.57	2.42	0.76	0.89	0.34	0.75	0.93	0.73	60	4

Table S3. Value of each comprehensive indicators (Z), subordinate function values, μ(X), comprehensive evaluation value (D) and order for inbred japonica rice varieties.

Type	Z1	Z2	Z3	Z4	Z5	U1	U2	U3	U4	U5	D	Order	Cluster
N0-22	0.65	0.84	-0.09	-0.20	1.74	0.76	0.77	0.54	0.32	0.82	0.66	56	1
N0-32	1.63	0.18	0.96	0.39	0.46	1.00	0.63	0.80	0.46	0.59	0.76	69	1
N0-34	1.21	0.98	-0.49	-0.38	2.24	0.90	0.80	0.44	0.28	0.91	0.70	65	1
N0-35	0.90	0.63	0.27	-0.45	0.15	0.82	0.73	0.63	0.26	0.53	0.66	54	1
N0-38	1.39	-0.07	-0.36	0.63	1.01	0.94	0.57	0.47	0.52	0.69	0.68	59	1
N150-22	0.60	0.60	0.37	0.35	0.11	0.75	0.72	0.65	0.45	0.52	0.67	57	1
N150-23	-0.23	1.88	0.09	1.18	1.20	0.56	1.00	0.58	0.65	0.72	0.69	64	1
N150-25	0.99	-1.15	0.95	1.42	1.31	0.85	0.34	0.79	0.71	0.74	0.68	61	1
N150-32	0.95	0.15	0.95	0.02	-0.45	0.84	0.62	0.79	0.37	0.42	0.68	58	1
N150-38	1.29	0.49	0.28	1.29	-0.35	0.92	0.70	0.63	0.68	0.44	0.74	67	1
N150-41	0.73	0.68	0.14	-0.03	0.48	0.79	0.74	0.60	0.36	0.59	0.66	55	1
N300-22	0.03	0.85	1.33	0.81	-0.33	0.62	0.78	0.88	0.56	0.44	0.69	62	1
N300-25	0.91	-0.67	1.59	1.78	0.87	0.83	0.44	0.95	0.79	0.66	0.74	68	1
N300-32	1.03	0.11	1.10	-0.26	-0.39	0.86	0.61	0.83	0.31	0.43	0.68	60	1
N300-33	0.77	0.49	1.39	-0.39	-0.49	0.79	0.70	0.90	0.27	0.41	0.69	63	1
N300-36	-0.31	1.08	0.93	2.66	0.40	0.54	0.82	0.79	1.00	0.57	0.72	66	1
N0-23	0.51	1.32	-0.12	-0.72	-0.47	0.73	0.88	0.53	0.20	0.41	0.63	49	2
N0-24	0.96	0.91	-0.25	-1.55	-0.98	0.84	0.79	0.50	0.00	0.32	0.60	43	2
N0-25	0.76	-1.32	0.38	0.29	2.13	0.79	0.30	0.66	0.44	0.89	0.60	44	2
N0-33	0.74	0.61	0.22	-0.55	-0.50	0.79	0.72	0.62	0.24	0.41	0.63	50	2
N0-40	0.20	0.96	-0.36	-0.38	0.42	0.66	0.80	0.47	0.28	0.58	0.60	41	2
N150-26	0.58	0.96	0.38	-0.66	0.36	0.75	0.80	0.65	0.21	0.57	0.65	53	2
N150-28	1.29	-2.01	0.60	0.46	-0.93	0.92	0.15	0.71	0.48	0.33	0.58	37	2
N150-30	-0.61	1.29	1.33	0.59	-1.93	0.46	0.87	0.89	0.51	0.15	0.63	48	2
N150-33	0.43	0.75	0.90	-0.78	-0.63	0.71	0.75	0.78	0.18	0.39	0.64	51	2
N150-34	0.67	0.98	-1.02	-0.18	0.62	0.77	0.80	0.31	0.32	0.61	0.61	46	2
N150-35	-0.20	0.47	0.47	0.26	0.18	0.56	0.69	0.68	0.43	0.53	0.60	40	2
N150-36	0.07	0.61	-1.84	2.03	0.90	0.63	0.72	0.11	0.85	0.67	0.58	38	2
N300-23	-0.37	0.91	0.61	0.97	-0.44	0.52	0.79	0.71	0.60	0.42	0.63	47	2
N300-26	-0.30	0.79	0.75	-0.50	0.37	0.54	0.76	0.74	0.25	0.57	0.59	39	2
N300-28	1.13	-1.73	0.50	1.03	-0.42	0.88	0.21	0.68	0.61	0.42	0.61	45	2
N300-31	-0.25	0.51	1.09	-0.15	-0.66	0.55	0.70	0.83	0.33	0.38	0.60	42	2
N300-41	0.36	0.78	0.25	0.05	0.36	0.70	0.76	0.62	0.38	0.57	0.64	52	2
N0-26	0.06	0.21	-0.32	0.38	-0.88	0.62	0.63	0.48	0.46	0.34	0.55	32	3
N0-28	1.61	-1.95	-1.12	-0.74	-0.77	1.00	0.17	0.29	0.19	0.36	0.49	23	3
N0-30	-0.01	0.48	-1.57	-1.04	0.80	0.61	0.70	0.18	0.12	0.65	0.48	19	3
N0-31	0.04	0.29	0.41	-1.14	-0.45	0.62	0.65	0.66	0.10	0.42	0.55	31	3
N0-36	0.21	1.06	-1.95	0.57	0.22	0.66	0.82	0.09	0.50	0.54	0.56	35	3
N0-41	0.83	-0.14	-0.58	-0.96	0.38	0.81	0.56	0.42	0.14	0.57	0.56	36	3
N150-24	-0.59	0.46	-0.56	-0.15	0.74	0.47	0.69	0.43	0.33	0.64	0.51	24	3

N150-29	0.89	-1.77	0.90	-0.78	0.32	0.82	0.20	0.78	0.18	0.56	0.55	33	3
N150-31	-0.11	0.08	0.82	-0.77	-0.56	0.58	0.61	0.76	0.18	0.40	0.55	34	3
N150-37	0.68	-1.37	-0.75	1.17	0.41	0.77	0.29	0.38	0.65	0.58	0.54	30	3
N150-40	0.38	0.27	-1.24	-1.50	-0.20	0.70	0.65	0.26	0.01	0.47	0.49	21	3
N150-42	0.05	0.77	-1.29	-0.19	-1.16	0.62	0.76	0.25	0.32	0.29	0.51	26	3
N300-27	-0.68	0.44	1.13	-1.15	-1.79	0.45	0.69	0.84	0.10	0.17	0.51	25	3
N300-30	-0.34	0.45	-0.19	-0.75	-0.03	0.53	0.69	0.51	0.19	0.50	0.51	27	3
N300-34	-0.15	0.47	-0.26	-0.39	0.19	0.57	0.69	0.50	0.28	0.54	0.54	29	3
N300-35	-0.93	0.21	0.91	-0.92	-0.62	0.39	0.63	0.78	0.15	0.39	0.49	22	3
N300-38	0.60	-0.57	-0.62	-0.09	-0.23	0.75	0.47	0.41	0.35	0.46	0.54	28	3
N300-43	-1.83	-0.10	1.51	0.87	-1.15	0.17	0.57	0.93	0.58	0.29	0.48	20	3
N0-27	-0.20	-0.77	-1.68	-1.04	-0.25	0.56	0.42	0.15	0.12	0.45	0.38	5	4
N0-29	1.03	-2.71	-0.77	-1.31	-0.49	0.86	0.00	0.37	0.06	0.41	0.41	8	4
N0-37	0.39	-1.71	-0.76	1.11	-2.73	0.70	0.22	0.38	0.63	0.00	0.46	18	4
N0-39	-1.14	-0.75	0.70	-1.54	0.52	0.34	0.43	0.73	0.00	0.60	0.41	9	4
N0-42	-0.59	0.55	-2.30	0.82	-0.45	0.47	0.71	0.00	0.56	0.42	0.44	15	4
N0-43	-1.69	-0.86	-1.14	-1.16	0.44	0.21	0.40	0.28	0.09	0.58	0.28	1	4
N0-44	-1.61	-1.09	-1.23	0.02	0.51	0.22	0.35	0.26	0.37	0.59	0.31	2	4
N150-27	-1.29	0.13	-0.20	-0.01	-1.23	0.30	0.62	0.51	0.36	0.28	0.43	13	4
N150-39	-1.77	-0.55	1.29	-1.00	2.20	0.19	0.47	0.88	0.13	0.91	0.44	14	4
N150-43	-2.16	-0.01	0.76	0.81	-1.71	0.09	0.59	0.75	0.56	0.19	0.41	10	4
N150-44	-1.97	-0.28	-1.09	2.20	-0.07	0.14	0.53	0.30	0.89	0.49	0.40	7	4
N300-24	-0.41	0.21	-2.00	-1.42	-0.32	0.51	0.64	0.07	0.03	0.44	0.38	6	4
N300-29	-0.33	-2.51	0.99	-0.20	-0.94	0.53	0.04	0.80	0.32	0.33	0.42	11	4
N300-37	-0.49	-1.47	-1.39	2.44	0.09	0.49	0.27	0.22	0.95	0.52	0.45	17	4
N300-39	-2.55	-1.46	1.80	-1.38	2.72	0.00	0.27	1.00	0.04	1.00	0.34	3	4
N300-40	-0.94	0.16	-0.22	-0.83	0.15	0.39	0.62	0.51	0.17	0.53	0.45	16	4
N300-42	-1.30	0.60	-0.87	0.11	-1.38	0.30	0.72	0.35	0.40	0.25	0.42	12	4
N300-44	-2.22	-0.62	-0.43	0.92	1.36	0.08	0.45	0.46	0.59	0.75	0.37	4	4

Table S4. Multiple regression analysis for determining correlations between comprehensive quality and 15 quality traits.

Rice type	Model	Unstandardised coefficients B	Standard error	Significance	F	R^2	Adjusted R^2
Hybrid indica rice	(Constant)	-2.241	0.065	0			
	Overall eating quality	0.004	0.001	0	130.0243	0.685	0.679
	Grain length	0.067	0.005	0	236.580	0.889	0.885
	Taste	0.01	0.001	0	396.513	0.954	0.951
	Smell	0.007	0.001	0	429.911	0.968	0.966
	Length/width	0.061	0.008	0	401.061	0.973	0.970
	Appearance	0.005	0.001	0	447.531	0.980	0.978
	Texture	0.006	0.001	0	792.753	0.990	0.989
Inbred japonica rice	(Constant)	-4.535	0.104	0			
	Head rice percentage	0.014	0.001	0	108.731	0.619	0.613
	Overall eating quality	0.011	0.001	0	166.131	0.834	0.829
	Mild rice percentage	0.021	0.002	0	221.328	0.911	0.907
	Chalkiness rae	0.001	0	0	250.957	0.940	0.936
	Grain length	0.14	0.011	0	26.0115	0.954	0.950
	Brown rice percentage	0.013	0.002	0	304.666	0.967	0.964
	Retrogradation	0.005	0.001	0	371.014	0.977	0.974
	Length/width	-0.183	0.022	0	688.956	0.989	0.998

Variables eliminated during regression are not included in the table. R2 and Adjust R2 comprise the sum of the accumulation of variables.
